# Systemic Corticosteroids Therapy in the Management of Acute Zonal Occult Outer Retinopathy

**DOI:** 10.1155/2015/793026

**Published:** 2015-01-11

**Authors:** San-Ni Chen, Chang-Hao Yang, Chung-May Yang

**Affiliations:** ^1^Department of Ophthalmology, Changhua Christian Hospital, Changhua City 500, Taiwan; ^2^School of Medicine, Chung Shan Medical University, Taichung 402, Taiwan; ^3^School of Medicine, Kaohsiung Medical University, Kaohsiung 807, Taiwan; ^4^Department of Ophthalmology, National Taiwan University Hospital, Taipei 100, Taiwan; ^5^School of Medicine, National Taiwan University, Taipei 10617, Taiwan

## Abstract

*Purpose*. To report the efficacy of systemic steroid in treating acute zonal occult outer retinopathy (AZOOR). *Methods*. Retrospective study of 9 consecutive patients of AZOOR, who received systemic steroid therapy in Changhua Christian Hospital from 2005 to 2013, is presented. The duration of therapy was at least 3 months. Patients were evaluated with best corrected visual acuity (BCVA), optical coherence tomography (OCT), fluorescein angiography (FA), indocyanine green angiography (ICG), visual field test, and electroretinography (ERG). *Results*. At the initial visit, visual field defect was noted in all patients and impaired visual acuity was noted in 4 eyes. OCT examination revealed disrupted ellipsoid zone at the macular area in 8 eyes and outer nuclear layer (ONL) loss in 1 eye. At the end of follow-up, all patients had improvement of visual field. The 4 eyes with initial impaired visual acuity had BCVA recovering to 20/20. Follow-up OCT showed partial or complete recovery of the ellipsoid zone at the macular area in the 8 eyes with initial disrupted ellipsoid zone and stable condition in the eye with ONL loss. The mean follow-up duration was 47.11 ± 26.65 months. *Conclusion*. Visual improvement was achieved in most cases of recent onset AZOOR after systemic steroid treatment.

## 1. Introduction

Acute zonal occult outer retinopathy (AZOOR) is a rare disease characterized by acute visual field loss accompanied with photopsia, absence or a minimal presence of vitreous cells, minimal fundus changes, normal fluorescein angiography, and decreased amplitudes of electroretinographic waves [1, 2]. Visual field defect often persists in patients with AZOOR [2]. Although the clinical presentations have been well documented, controversy remains about the treatment of AZOOR. Here we report the therapeutic effect of corticosteroid in a consecutive case series of patients with recent onset AZOOR.

## 2. Patients and Method

This was a retrospective review of 9 consecutive patients diagnosed with AZOOR receiving corticosteroid therapy from 2005 to 2012. The study was carried in adherence to the Declaration of Helsinki and was under the approval of Institutional Review Board in Changhua Christian Hospital. Only those patients of primary AZOOR with disease onset of less than 3 months were included. The diagnosis of AZOOR was based on the following criteria: acute visual field defect with or without photopsia; no abnormal fundus and disc changes except myopic fundus tessellation; no definite leakage or staining in fluorescein angiography (FA) aside from mild segmental perivenous staining indicating phlebitis; and reduced waves in multifocal and/or full-field electroretinography (mfERG/ffERG). The diagnosis was in accordance with the original strict inclusion and exclusion criteria reported by Gass [1, 2]. Granular fovea, white dots, or punctate lesions were particularly searched for in suspicious cases to rule out other possible disease entities, such as multiple evanescent white dot syndrome (MEWDS) or punctate inner choroidopathy (PIC). If initially included patients developed fundus lesions compatible with PIC, multifocal choroiditis (MFC), MEWDS, acute macular neuroretinopathy (AMN), or others in the spectrum of white dot syndrome [3] during the follow-up period, they were also excluded. All patients underwent ophthalmologic work-up including best corrected visual acuity (BCVA), slit lamp biomicroscopy, indirect fundus ophthalmoscopy, optical coherence tomography (OCT, Stratus OCT, Carl Zeiss, before January 2009; Cirrus OCT, Carl Zeiss, after January 2009), visual field test (Humphrey Field Analyzer, Carl Zeiss Meditec, Dublin, CA, USA, 30-2 SITA program), full-field electroretinography (ffERG) and multifocal ERG (mfERG), fundus autofluorescence (FAF), and FA and indocyanine green angiography (ICG) at the time of diagnosis and regularly during the follow-up period. The follow-up time was at least two years.

## 3. Results

**Figure 1:**
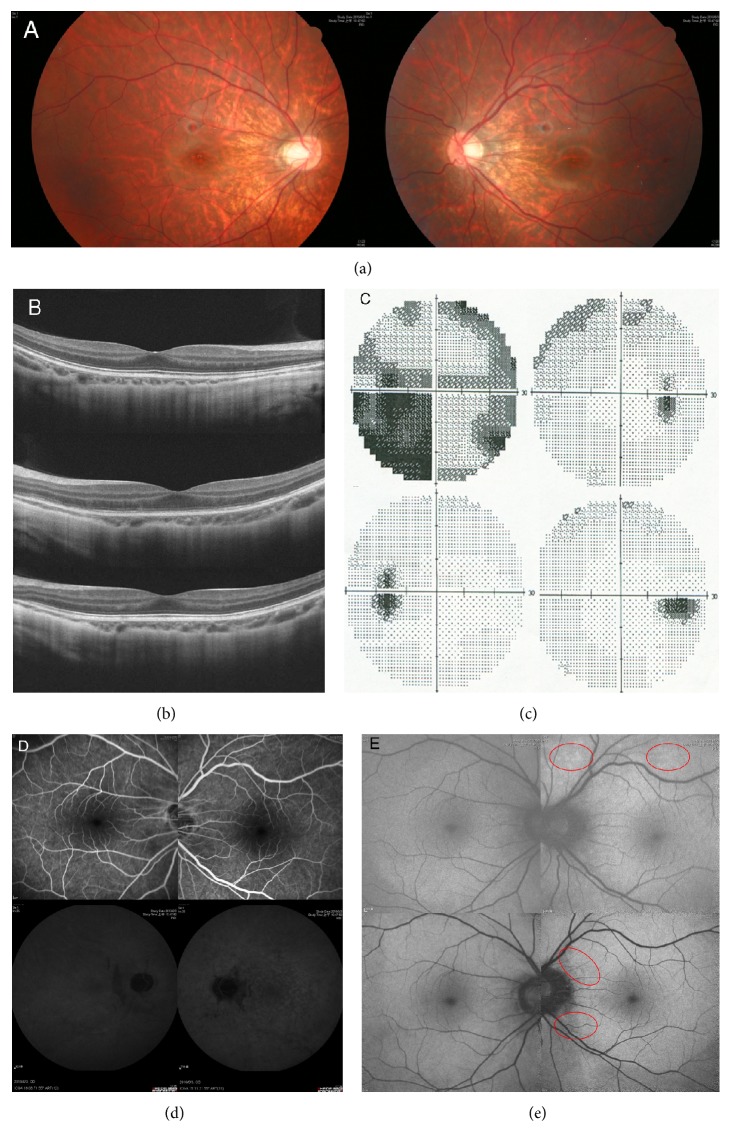
Color fundus in case 3 showed a tessellated myopic fundus with temporal conus in both eyes (a). Optical coherence tomography showed a normal picture in the right eye (b, upper) and a disrupted ellipsoid zone in the left eye at the acute onset stage (b, middle). Recovery of the ellipsoid zone in the left eye was noted 12 months later (b, lower). Humphrey 30-2 visual field showed generalized depressed light sensitivity and visual field defect at the central, temporal lower, and circumferential periphery in the left eye (c, upper). Mild obliteration at the nasal and upper periphery was also noted in the right eye (c, upper). Visual field test 1 year later showed an almost completely recovered visual field in the left eye (c, lower). Fluorescein angiography at the acute stage did not reveal any abnormalities (d, upper). Indocyanine green angiograph shows multiple hypofluorescent, coalescent spots at the posterior pole and peripapillary area in the left eye (d, lower). Fundus autofluorescence (FAF) at acute phase showed some hyperautofluorescent spots at upper aspect of disc and upper vascular arcade in the left eye (e, upper, red circle). FAF imaging 1 year later showed some peripapillary mottled hypoautofluorescence (e, lower, red circle).

**Figure 2:**
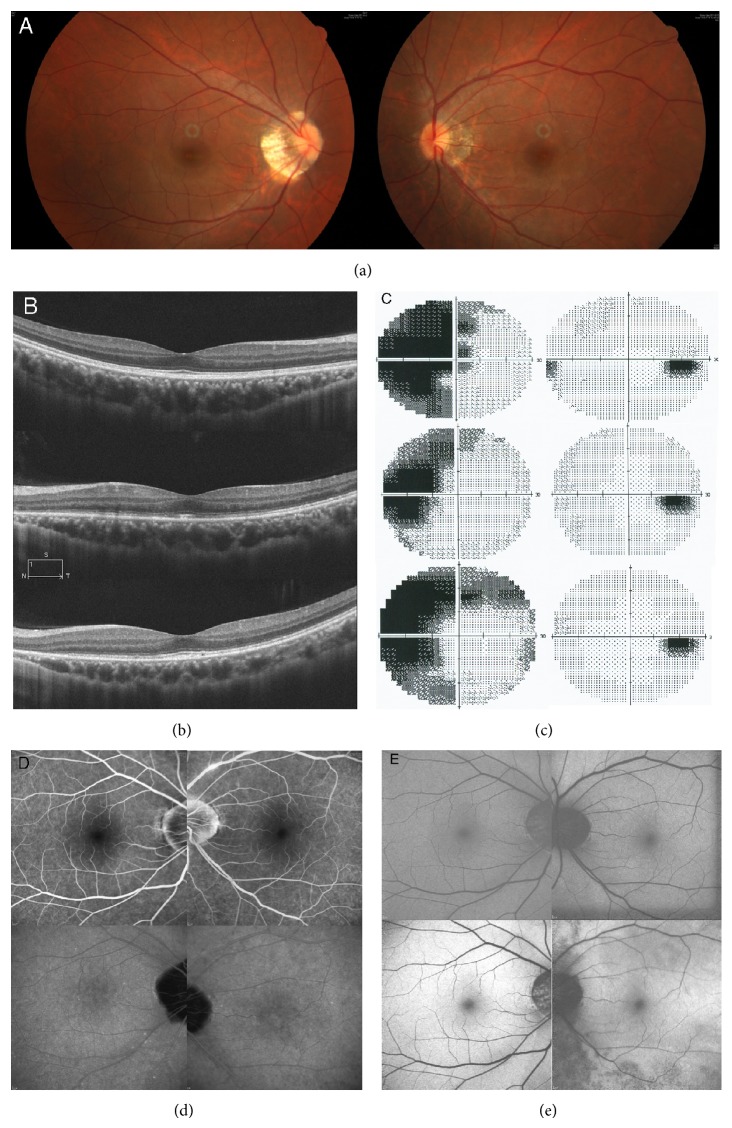
Color fundus in case 5 shows tessellated fundus in both eyes with temporal conus (a). Optical coherence tomography showed normal ellipsoid zone in the right eye (b, upper) and disrupted ellipsoid zone at the macular and peripapillary area with central sparing in the left eye (b, central) in the acute stage. Recovery of the ellipsoid zone was noted at the macular area 4 months later; however loss of the outer nuclear layer and the ellipsoid zone adjacent to the disc was also noted (b, lower). Visual field test at initial presentation showed dense central, temporal scotoma, and faint circumferential peripheral visual field defect in the left eye (c, upper). Small nasal scotoma and faint upper nasal scotoma were also noted in the left eye. The visual field defect in the left eye reduced a little 3 months later after oral prednisolone treatment (c, middle). An enlarged visual defect was noted after tapering of the steroids (c, lower). Fluorescein angiograph showed mild segmental periphlebitis in the left eye at the disease onset (d, upper). Indocyanine green angiography taken concurrently revealed some suspicious hypofluorescent spots at the posterior pole and arcade area in the left eye (d, lower). Fundus autofluorescence (FAF) imaging at acute phase showed some suspicious hyperautofluorescent spots at upper aspect of disc (e, left). Follow-up FAF 2 years later showed diffuse hypoautofluorescence at peripapillary area and area lower to the lower vascular arcade (e, right).

Nine patients (9 eyes) including 1 male and 8 females were included in this study. All study patients were myopic. The average age was 32.67 ± 11.18 years. The mean refractive status was −8.72 ± 4.81 D. The mean interval between symptom onset and clinic visit was 18.2 ± 23.95 days. The mean follow-up duration was 47.11 ± 26.65 months. The chief complaints at the initial visit included visual field defect in all 9 patients, photopsia in 8 patients, and impaired visual acuity in 4 patients. In the latter 4 patients, the initial BCVA was 20/1000 in one, 20/50 in another, and 20/40 in two; the other 5 eyes had initial BCVA of 20/20 or more. Fundus examination revealed tessellated fundus in all but 1 patient. Humphrey 30-2 visual field test revealed temporal scotoma, blind spot enlargement (BSE), and paracentral scotoma in all eyes; concentric visual field defect with only small central island left in 2 eyes; arcuate scotoma in 1 eye; central scotoma in 1 eye (case 5); and peripheral scotoma (other than the temporal half, beyond central 20 degrees) in 4 eyes. Visual field abnormalities were noted in 9 fellow eyes. The mean loss of visual field at the first visit was −15.37 ± 7.07 dB. OCT showed ellipsoid zone disorganization at the macular and juxtapapillary area in 8 eyes (Figures 1(b) and 2(b)). The remaining eye showed the disappearance of the outer nuclear layer (ONL) from the optic disc to the parafoveal area at the initial visit (case 4). No particular findings of OCT were noted in all the fellow eyes at the macular area. FAF imaging showed mottled autofluorescence at peripapillary area or around the vascular arcades in 6 cases (Figure 1(e)). FA was unremarkable in 8 eyes. Mild segmental phlebitis was noted in 1 eye (Figure 2(d)). Indocyanine green angiography showed multiple partially confluent hypofluorescent patches at the posterior pole in one eye (Figure 1(d)) and unremarkable ones in the other eyes. Reduced amplitude in ffERG and mfERG was noted in all eyes.

Three patients (cases 3, 8, and 9) had intravenous pulse steroid therapy (methylprednisolone 250 mg/q 6 h for 3 days) as the initial treatment followed by oral prednisolone with gradual tapering within 3 months. Five patients (cases 1, 2, 4, 6, and 7) received oral prednisolone 1 mg/kg/day as the initial treatment with gradual tapering in the following 3 months. One patient (case 5) had oral prednisolone 1 mg/kg/day initially, but the medication could not be successfully tapered because of the reactivation of the disease. She was then maintained on oral prednisolone 10 mg per day and sodium mycophenolate 360 mg bid during the 3 years of follow-up.

At the final follow-up visit, all of the patients had improvement in visual field. The mean visual field loss improved from −15.37 ± 7.07 dB at initial visits to −5.64 ± 3.01 dB at the final visits (*P* = 0.009, paired *t*-test). The 3 patients (cases 3, 8, and 9) receiving pulse therapy had excellent visual field recoveries, all better than the −5 dB visual field loss (Figure 1(c)). The case with long-term immunotherapy and maintenance dose of prednisolone (case 5) had the least visual field recovery. This patient suffered from visual field defect progressing on tapering of oral steroid (Figure 2(c)). Follow-up OCT showed partial or total recovery of the ellipsoid zone at the foveal and perifoveal areas in all the 8 eyes with initial disrupted ellipsoid zone (Figures 1(b) and 2(b)). In the case with long-term prednisolone and immunosuppressants (case 5), loss of ONL at peripapillary was noted (Figure 2(b)). Follow-up FAF showed minimal hypo-FAF patches at the peripapillary area in 7 eyes (Figure 1(e)). The other 2 eyes (cases 4, 5) had obvious FAF changes (Figure 2(e)), and both of them had ONL loss either at the initial presentation (case 4) or during the follow-up period (case 5). Recurrent disease was noted in two eyes (cases 6 and 7) at 2 and 5 years later, respectively, and 3 cases (cases 1, 2, and 5) experienced deterioration of the visual field after stopping or tapering the systemic steroid. The demographic data of the patients are listed in Table 1.

## 4. Discussion

The pathogenesis of AZOOR is still unclear. Infectious agents, such as virus [4] and autoimmunity [5], had been raised as the possible etiologies of AZOOR. Jampol and Becker proposed that there might be interplay between non-disease-specific gene and environmental triggers, including virus infection, stress, and personal factors such as sex, and other genes, resulting in the development of different diseases in AZOOR complex [6].

Natural course of AZOOR is variable. In the report of Gass et al., only 26% of cases showed variable improvement and 19% of cases showed deterioration [4]. Most cases have visual field stabilized 6 months after the disease onset. In those eyes with vision improvement, only 5% had AZOOR related fundus changes; in contrast, in those without vision improvement, as many as 66% of eyes had AZOOR related fundus changes. Recently, Mrejen et al. described the characteristic features of AZOOR, which included a demarcating line of the progression at the level of the outer retina; a trizonal pattern of sequential involvement of the outer retina, the retinal pigment epithelium, and the choroid; and frequent zonal progression [7]. Since most of the RPE and choroidal changes are sequentially involved, we believe they are secondary changes following the death of photoreceptors cells, just like the disease processes in retinitis pigmentosa, in which RPE and choroidal changes occur after the death of photoreceptors. Thus it is reasonable to postulate that if, after the acute episodes of AZOOR attack, the photoreceptors can recover without permanent damage by more aggressive treatment, those secondary changes may be minimized. This postulation was supported by the clinical manifestations of our cases, in which while most eyes had only limited FAF changes, the 2 eyes with marked FAF changes during the follow-up period either had ONL loss at initial presentation (case 4) or had persistent visual field defect and sequential permanent photoreceptor loss (case 5). Compared to the outcome in other series [4], the better results in our series may be attributed to the fast initiation of the steroid therapy at the acute or subacute stage of the disease, which salvaged the photoreceptors from permanent damage. In addition, all the 3 patients (cases 2, 8, and 9) having pulse steroid therapy as the initial treatment had excellent recovery (all had final visual field loss less than 5 dB) and their visual field kept improving over 6 months after the disease onset. Our treatment results indicate that AZOOR is an inflammatory disease and aggressive systemic steroid therapy at an early stage may better reverse the natural course of AZOOR.

The therapeutic effects of systemic steroid or immunosuppressive agents had been sporadically reported in the literatures [8, 9]. Spaide et al. had reported 2 cases of AZOOR which showed visual field improvement after oral prednisolone and immunomodulation agents [8]. Kitakawa et al. also reported dramatic improvement of VA and visual field after pulse steroid therapy in a patient with AZOOR [9]. Steroid therapy has also been shown to be effective in other inflammatory diseases involving the outer retina. In the report of Chen and Hwang [10], 4 eyes with PIC and zonal outer retinopathy showed great improvement in VA, visual field test, and ellipsoid zone in OCT after systemic steroid therapy soon after the disease onset. Administration of systemic steroid of Vogt-Koyanagi-Harada disease at the acute stage has long been known to help prevent permanent visual loss [11]. Eyes with serpiginous choroiditis [12] also benefit from steroid therapy in the early stage of the disease. Though VKH disease, serpiginous choroiditis, and PIC with zonal outer retinopathy are different disease entities from AZOOR, the rapid anti-inflammatory effect of steroid may help reverse the catastrophic consequences of the fragile outer retina inflicted by the inflammation. The controversy on the effect of corticosteroid in AZOOR may be partly due to the frequently delayed diagnosis of AZOOR in the past before spectral domain OCT was available. A delayed treatment may not help outer retinal tissue recovery in time, and the tissue damage may have already passed over the point of return. In this study, most of our patients had AZOOR diagnosed and treated within a short time after the disease onset (most of them within 2 weeks). This may explain why almost all our cases responded favorably to corticosteroid therapy.

## 5. Conclusion

In summary, systemic corticosteroid therapy seems to be beneficial in changing the visual outcome of AZOOR. The limitations of this study are the retrospective nature, small cases' number, and lack of control group. Further studies are necessary to further validate the conclusion.

## Figures and Tables

**Table 1:** Demographic data of patients.

No./age (y)/sex	Refractive status (D)	Onset duration	Treatment	Initial VA	Final VA	Initial/final VF loss (dB)	F/U (m)
1/45/F	−5.5	2 w	Oral pred.	20/20	20/20	−8.85/−6.61	24
2/35/F	−10.0	4 d	Oral pred.	20/20	20/20	−8.27/−5.07	42
3/18/M	12.75	1 d	IV pulse → oral pred.	20/2000	20/20	−15.27/−2.90	48
4/12/F	−2	2 m	Oral pred.	20/20	20/20	−11.79/−5.01	45
5/30/F	−10.0	4 d	Oral pred. + myco.	20/20	20/20	−16.85/−12.76	37
6/41/F	−16.0	1 w	Oral pred.	20/40	20/20	−10.30/−6.42	61
7/30/F	−10.5	1 w	Oral pred.	20/50	20/20	−21.47/−6.05	111
8/33/F	−1.5	2 m	IV pulse → oral pred.	20/20	20/20	−26.49/−2.51	30
9/50/F	−10.25	1 w	IV pulse → oral pred.	20/40	20/20	−13.42/−3.5	26

No.: patient number; y: year; M: male; F: female; D: diopter; d: day; m: month; oral pred.: oral prednisolone starting with 1 mg per Kg of body weight; IV pulse: intravenous pulse of methylprednisolone; myco.: sodium mycophenolate; VA: Snellen visual acuity; dB: decibel; and F/U: duration of follow-up.
